# A comparison of multiple shRNA expression methods for combinatorial RNAi

**DOI:** 10.1186/1479-0556-9-9

**Published:** 2011-04-17

**Authors:** Glen J Mcintyre, Allison J Arndt, Kirsten M Gillespie, Wendy M Mak, Gregory C Fanning

**Affiliations:** 1 Johnson and Johnson Research Pty Ltd, Level 4 Biomedical Building, 1 Central Avenue, Australian Technology Park, Eveleigh, NSW, 1430, Australia; 2 School of Biotechnology and Biomolecular Sciences, The University of New South Wales, Sydney, NSW 2052, Australia; 3 Tibotec BVBA, Gen De Wittelaan L 11 B3, 2800 Mechelen, Belgium

## Abstract

RNAi gene therapies for HIV-1 will likely need to employ multiple shRNAs to counter resistant strains. We evaluated 3 shRNA co-expression methods to determine their suitability for present use; multiple expression vectors, multiple expression cassettes and single transcripts comprised of several dsRNA units (aka domains) with each being designed to a different target. Though the multiple vector strategy was effective with 2 shRNAs, the increasing number of vectors required is a major shortcoming. With single transcript configurations we only saw adequate activity from 1 of 10 variants tested, the variants being comprised of 2 - 3 different target domains. Whilst single transcript configurations have the most advantages on paper, these configurations can not yet be rapidly and reliably re-configured for new targets. However, our multiple cassette combinations of 2, 3 and 4 (29 bp) shRNAs were all successful, with suitable activity maintained in all positions and net activities comparable to that of the corresponding single shRNAs. We conclude that the multiple cassette strategy is the most suitably developed for present use as it is easy to design, assemble, is directly compatible with pre-existing shRNA and can be easily expanded.

## Introduction

The recently discovered RNA interference (RNAi) pathway is a post-transcriptional gene silencing and regulation mechanism with potential application in the field of gene therapy. In mammalian cells RNAi begins with a double-stranded RNA inducer that is progressively processed from its termini by RNase III type endonucleases, firstly Drosha in the nucleus followed by Dicer in the cytoplasm, to yield a short interfering RNA (siRNA) duplex of ~ 22 bp [1,2]. The duplex is unwound and loaded into the RNA induced silencing complex (RISC) in a process that favors one of the two strands (the guide strand) based on a difference in thermodynamic stability at the ends of the duplex [3]. The most common natural substrates for mammalian RNAi are microRNA, short hairpin-like RNA transcripts implicated in regulating gene expression activity [1,2]. The RNAi pathway can be artificially engaged at any point in the process, typically either through delivering synthetic siRNAs to the RISC [4,5] or by expressing short hairpin RNAs (shRNA or hairpins) to be processed by Dicer and possibly Drosha [6,7].

shRNAs are well suited for use in current gene therapy plans. shRNA consists of a short single-stranded RNA transcript that folds into a 'hairpin' configuration by virtue of self-complementary regions separated by a short 'loop' sequence. Whilst hairpins can be expressed from either polymerase (pol) III or more recently pol II promoters, it is the U6 and H1 pol III promoters that have been most extensively employed owing in part to their relatively well-defined transcription start and end points [6,7]. Importantly, pol III based hairpin expression cassettes have been incorporated into viral vectors which have been stably integrated both in culture and whole animals with effective silencing maintained over time [8-10]. The potency of individual shRNA directed to HIV has been extensively demonstrated [11-13], however, several studies have also shown single shRNA can be rapidly overcome by the emergence of escape mutants [14-17]. Modeling shows that perhaps as few as 4 shRNAs used in combination may be sufficient to prevent the emergence of escape mutants [18-22]. This idea is supported by several wet studies showing that in laboratory conditions HIV-1 escape can be delayed by using more than one shRNA [11,23-26]. In a clever variation on this idea, some have designed anticipatory shRNAs specifically to block known escape routes, though when tested it was found that the virus still evolved around these [27,28]. There is now clearly a need for an evaluation of multiple shRNA expression strategies to identify those that can be readily integrated into current anti-HIV gene therapy research programs.

One method for expressing multiple shRNAs is to use separate expression vectors encoding individual shRNA. Multiple shRNAs have been used successfully against cellular, viral and exogenous gene targets by the use of either multiple plasmid [29], retrovirus [30] or lentivirus vectors [9]. Multiple shRNAs can also be combined into a single expression vector via several self-contained expression cassettes (e.g. 1 cassette = promoter, shRNA and terminator), of which there are now many examples [11,31-34]. Alternatively, multiple shRNA domains can be combined in a single transcript, of which there are two base configurations; distinct hairpin domains joined 3' to 5' in what we call a 'cluster' (CL) configuration, and a 'head-to-tail' (HT) configuration in which all the sense stem regions are joined first, followed by a single loop and then all the anti-sense regions [17,35-39]. This second configuration appears as a long hairpin which, depending on the design, may be punctuated by unpaired spacer regions between the hairpin domains. Single transcript strategies are the most compact, and in this respect the most desirable means of co-expressing multiple shRNAs for gene therapy, but with few examples and no design guidelines yet reported, its general ease-of-use is unclear.

The aim of this study was to evaluate the 3 different shRNA co-expression methods to determine their suitability for present use in gene therapy schemes, with a key focus on ease of construction, applicability to new sequences, and the retention of suppressive activity in the component shRNAs. We assembled combinations of 2 to 4 shRNAs using 3 different strategies: one using multiple expression vectors, one using multiple hairpin expression cassettes and the other based on a single transcript comprised of different hairpin domains. While we were able to achieve successful suppression with each strategy, we concluded that the multiple cassette strategy is currently most useful due to ease of design, assembly, and its immediate compatibility with pre-existing shRNA already selected for high activity.

## Results

### Co-expression using multiple vectors

The effects of co-expressing hairpins of different sequence were first examined in its simplest form; using two hairpins, each expressed from a separate plasmid vector (**pS**) derived from pSilencer 3.0-H1 (Ambion). In this circumstance the suppressive activities of each hairpin were measured when separately expressed, and then again when co-expressed. All hairpins in this study were expressed from the human H1 polymerase III promoter. This experiment employed two 29 bp shRNAs against the HIV-1 genes encoding for Tat and Vif: Tat 56-29 (**T**) and Vif 88-29 (**V**) (variant NL4-3, accession #AF324493) (Table 1). Suppressive activity was measured by flow cytometry as a reduction in fluorescence of an appropriate reporter after transfection and transient expression of both hairpin(s) and reporter(s) in HEK293a cells. In this and all subsequent experiments the activity of each shRNA vector was measured relative to the activity of the appropriate control vector containing an equivalent number of 'empty' (**e**) expression cassettes (i.e. consisting of a promoter(s) plus terminator but expressing no hairpin(s)).

**Table 1:** shRNA sequences

r ^a^	Target	In. ^b^	Stem (sense)	Loop	Te. ^b^	**Len**.
**T**	Tat 56-29	G	AAACUGCUUGUACCAAUUGCUAUUGUAAA	ACTCGAGA	G	70
**V**	Vif 88-29	G	UAUAUUUCAAGGAAAGCUAAGGACUGGUU	UCTCGAGU	-	69
**R**	Vpr 72-29	-	GGAACUUAAGAGUGAAGCUGUUAGACAUU	ACTCGAGA	-	68
**U**	Vpu 158-29	-	GCAAUGAGAGUGAAGGAGAAGUAUCAGCA	ACTCGAGA	-	68
**O'**	Off #1-29	-	AAGACAGUCCAACACACGCCACCUGUCUC	UCTCGAGU	-	68
**O"**	Off #2-29	-	AACAGUCUGUCAAAGGUGACCCCUGUCUC	UCTCGAGU	-	68
**T^19^**	Tat 59-19	-	CUGCUUGUACCAAUUGCUA	UUCUCGAGA	-	50
**V^19^**	Vif 98-19	-	GGAAAGCUAAGGACUGGUU	UCTCGAGU	-	48

**^a ^**: shRNA abbreviation

**^b ^**: In. (**In**itiating)/Te. (**Te**rminating): Extra nucleotides included 5'(the first nt. transcribed) and 3' (the last nt. transcribed prior to 1 or more U termination remnants) in only some sequences (those which were also a part of an overlapping study).

**Figure 1:**
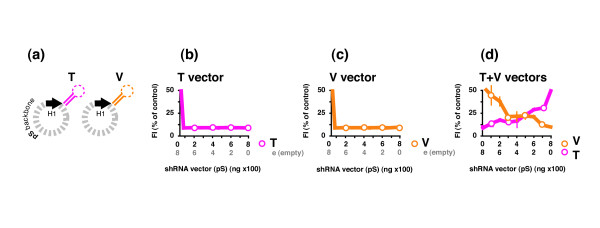
**Activities of different shRNAs from different vectors**. (**a**) The first two test hairpins, Tat 56-29 (**T**) and Vif 88-29 (**V**), were expressed from separate plasmid vectors (**pS**) via the H1 polymerase III promoter. HEK293a cells were transfected with 800 ng of the pSilencer-based (**pS**) T vector (+ empty) (**b**), the V vector (+ empty) (**c**), or the T and V vectors together (**d**) at mass ratios of 1:7 to 7:1, plus 100 ng each of both target-specific reporter vectors GFPs**T**at (T.r) and AsRed1s**V**if (V.r-red). There were 9 data points plotted for each curve, though for clarity open circles are only shown on every 2nd one. The data shown is representative of several replicated experiments (repeated twice, i.e. n = 2).

Either the two hairpin vectors, or each hairpin vector and the equivalent control vector were co-transfected at different ratios (Figure 1). The total amount of DNA delivered for each transfection was kept constant and the shRNA expression vector was always present at a level such that the RNAi process would presumably be saturated so that the effects of competition, if any, would be evident [40]. As each hairpin was directed to a different target the suppressive activity was measured using two unique reporters (GFPsTat and AsRed1sVif), which could be detected both simultaneously and independently. The specific activity of each hairpin vector was unaffected in the presence of empty expression vector at all ratios. However, the specific activity of each hairpin when co-expressed was progressively reduced at ratios that increasingly favored the competing hairpin. We surmise that reduction in specific activity was not due to a second vector (or promoter), but rather from inter-hairpin competition for access to the RNAi machinery. In summary, co-expressed hairpins delivered via separate vectors can function simultaneously, but do so at reduced levels due to competitive access for the RNAi machinery.

### Co-expression using multiple-cassette vectors: establishing positional effects

**Figure 2:**
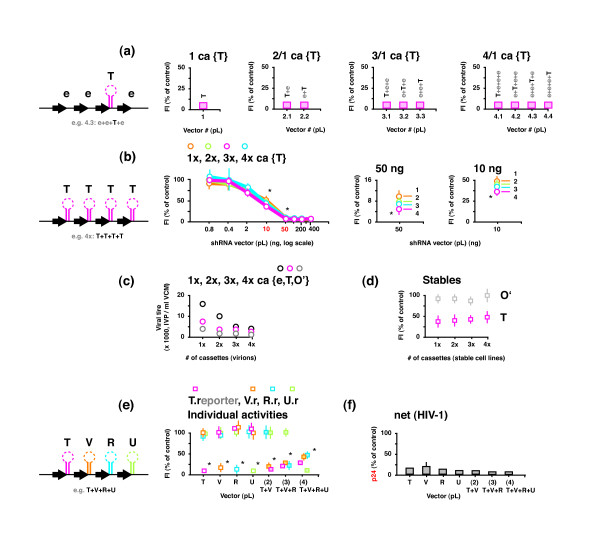
**Activities of individually expressed shRNAs from the same vector**. (**a**) 400 ng each of a series of 1, 2, 3 and 4 cassette (**ca**) pLenti6 (**pL**) based vectors with only a single hairpin per vector (surrounded by 'empty' expression cassettes) were assayed with 300 ng each of T.r (target-specific) and V.r-red (control) (n = 3). (**b**) 0 - 400 ng each of a series of 1×, 2×, 3× and 4× cassette pL vectors with all cassettes containing a T shRNA were assayed with 300 ng each of T.r and V.r-red. Total DNA delivered was kept constant at 1 μg by supplementing samples (as required) with the corresponding empty control vector. Statistically significant improvements in suppressive activity (*; P < 0.05) at 10 and 50 ng are shown at a finer scale (comparing the single cassette vector to the 2, 3, and 4 cassette vectors). (n = 3) (**c**) Viral titres of the Infectious virus produced from the 1×, 2×, 3× and 4× all T cassettes vectors, and the corresponding off-target (**O'**) and empty ("**e**") series (n = 1). (**d**) Stably integrated HEK293a cell lines for the 1× - 4× T, O' and e cassette vectors were assayed with 300 ng each of T.r and V.r-red, plus 400 ng of the appropriately matched 1 - 4 e cassette vector (to maintain a constant transfection amount of 1 μg) (n = 1). (**e**) 400 ng each of single shRNA vectors and a series of 1, 2, 3 and 4 cassette vectors made from 4 different shRNAs (T, V, R, and U) were separately assayed with 300 ng each of the 4 corresponding GFP fusion reporters T.r, GFPs**V**if (V.r), GFPsVp**r **(R.r) and GFPsVp**u **(U.r) plus 300 ng of an AsRed1s**N**ef control (N.r-red). (*) There was a statistically significant reduction in the individual suppressive activities of each hairpin relative to their single counterparts (P < 0.01) (n = 1). (**f**) Net suppressive activities of the same vectors were measured using a HIV-1 production assay by transfecting HEK293a cells with 110 - 130 ng of shRNA vector with 800 ng of pNL4-3 reporter and measuring the impact on p24 production (n = 2).

An alternative strategy to overcome the obvious limitations of using multiple vectors to co-express hairpins (e.g. issues of vector multiplicity) was to incorporate multiple hairpin expression cassettes within a single vector. However, to address concerns of potential promoter or transcriptional interference [41] it was important to first determine if each individual cassette position in a multiple cassette vector was capable of expressing a hairpin that functioned with equivalent suppressive activity. At this point we switched to using a pLenti6 derived vector backbone (**pL**) (Invitrogen) so that we could later test our constructs in stably transduced scenarios. We assembled a series of 1, 2, 3 and 4 cassette vectors containing only a single hairpin (**T**) expression cassette per vector, which was placed at each different position. The surrounding positions consisted of 'empty' expression cassettes, e.g. the 3 cassette vectors included 3.1: **T+e+e**, 3.2: **e+T+e**, and 3.3: **e+e+T**. There was ~ 130 bp of spacer sequence between each cassette, as measured from the terminator of one cassette (n) to the promoter of the next downstream cassette (n+1). Control vectors were also created that were of corresponding sizes and were composed of an equivalent number of all empty expression cassettes (2 to 4). Suppressive activity was first measured by flow cytometry as a reduction in fluorescence after transfection and transient expression from both hairpin and reporter vectors (Figure 2a). There was no apparent reduction in activity from any cassette position, from either the 2, 3 or 4 cassette vectors, with each cassette position retaining full hairpin activity equivalent to the single position, single hairpin cassette vector.

### Co-expression using multiple-cassette vectors: multiplication of a single hairpin

pLenti6 based vectors with 2, 3, and 4 cassettes were constructed with an identical hairpin (**T**) expression cassette placed in all positions to investigate whether increasing the cassette number could increase the suppressive activity (e.g. 2×: **T+T**, 3×: **T+T +T**, and 4×: **T+T+T+T**). Suppressive activity was measured across a range of vector amounts (from 400 - 0 ng) so that the effects of increasing cassette number could be investigated during both RNAi-saturating and sub-saturating conditions (Figure 2b). The total amount of DNA delivered for each transfection was kept constant by supplementing each reaction with the appropriate amount of corresponding control vector whilst keeping the amount of reporter vector constant. There were no differences in the suppressive activities from 400 ~ 100 ng of each vector delivered, which supported the hypothesis that the RNAi process was saturated across this range. However, at 50 - 10 ng of vector(s) there were statistically significant improvements in suppressive activity (*) with increasing cassette numbers (P < 0.05, comparing the single cassette vector to the 2, 3, and 4 cassette vectors). The trend did not extend below this concentration range, as effective suppressive activity was lost and accordingly any meaningful difference between the different numbers of cassettes.

We further examined whether multiplying an identical expression cassette would be beneficial in stably transduced cell lines. Infectious virus was generated from each of the 1, 2, 3 and 4 (**T**) cassette vectors, along with the empty control vectors, and another corresponding series of off-target shRNA cassette vectors (using an unmatched, off-target, 29 bp hairpin (**O'**) in 1, 2, 3, and 4 cassette combinations). Interestingly, when preparing virus, we saw a steady decrease in titre that correlated with increasing cassette number for both hairpin containing and empty expression cassette vectors (Figure 2c). We infected (transduced) HEK293a cells, selected for stable integrants, and measured suppressive activity by transfecting and transiently expressing the relevant reporter vectors into each cell line (Figure 2d). Analysis of the off-target hairpin controls showed no deleterious impact on fluorescence activity from the vector backbone, promoters or irrelevant hairpins. However, the suppression levels from the Tat-specific shRNA cell lines were reduced by ~ 30 - 40% relative to the maximum levels observed during transient expression (cf. Figure 2b), which was not unexpected and most likely due to low-copy number integration with a corresponding reduction in shRNA expression [8,18,42]. In this stable expression system there were no statistically significant differences in suppressive activity between the 2, 3 and 4 cassette cell lines.

### Co-expression using multiple-cassette vectors: diversifying hairpin targets

The multiple cassette strategy was further investigated by combining four different shRNA in a single vector using the same pL base vectors and cassette configurations as before. In addition to Tat 56-29 and Vif 88-29, two further (HIV-1) 29 bp shRNA were used: Vpr 72-29 (**R**) and Vpu 158-29 (**U**). Each shRNA was placed in both single cassette vectors and combination vectors of 2 (**T+V**), 3 (**T+V+R**) and 4 (**T+V+R +U**) cassettes. All vectors were assayed in turn with each of the relevant reporter vectors enabling the specific activity of each shRNA to be measured independently (Figure 2e). Each individual hairpin exhibited potent and specific activity for its matched assay vector(s). There was, however, a progressive and statistically significant reduction in the individual suppressive activity of each hairpin when expressed in combinations of increasing number, relative to the corresponding individual shRNA (P < 0.01), the exception being Vpu 158-29, which appeared unaffected. The activity of each vector was further examined using HIV-1 as the target and p24 production (a capsid protein) as the readout (Figure 2f). Whilst each hairpin was of a different sequence, their activities were now measured via a common endpoint (p24 production) and thus each could be considered as being directed towards a "common or shared target". The net suppressive activity of each multiple hairpin vector was similar to the corresponding individual hairpin vectors (all within 5% of each other). We concluded that whilst hairpins compete with each other for access to RNAi machinery, a net suppressive activity can be maintained from multiple simultaneously acting shRNAs.

### Co-expression using single transcript arrays of hairpin domains

**Figure 3:**
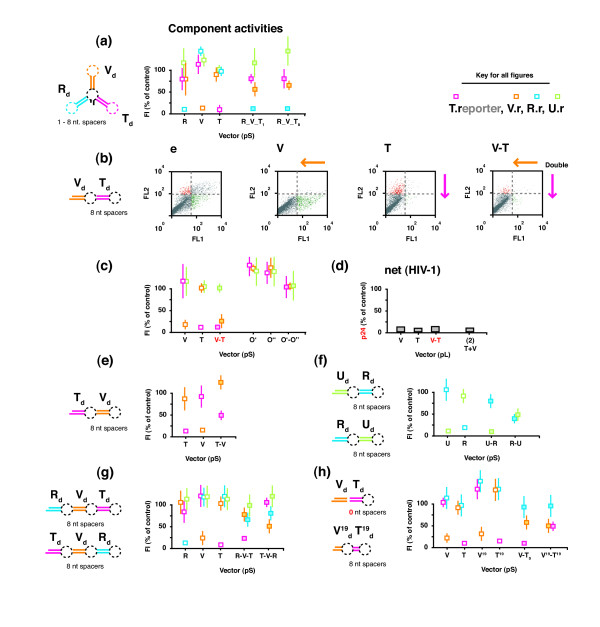
**Activities of multiple-domain, single transcript constructs**. (**a**) Two cluster configurations of R, V and T shRNA domains with (R_V_T_1 _and R_V_T_8_) and the corresponding single shRNA vectors were assayed using 200 ng of vector plus 400 ng each of R.r, V.r, T.r, and U.r plus N.r-red. The target domains are indicated schematically by: {*target*}_d_. (n = 2) (**b**) Flow cytometry dot plots from 200 ng of a head-to-tail configuration of the V and T shRNA stem regions (V-T) assayed with 400 ng each of the V.r-red and T.r reporters, showing channel-specific knockdown. (**c**) The same configuration was assayed with the Vr., T.r and U.r reporters and compared against a similar O'-O'' (control) configuration (similarly assayed) (n > 6). (**d**) The net activity of the V-T configuration (now expressed from a pL vector) was assayed using 110 - 130 ng of V-T vector and 800 ng of pNL4-3 reporter. (**e - h**) The component activities of further configurations, T-V, U-R, R-U, R-V-T, T-V-R, V-T_0_, V^19^-T^19 ^were similarly assayed using 400 ng of each plus 300 ng of one of the R.r, V.r, T.r, and U.r reporters and 300 ng of N.r-red (as indicated by the appropriately colored boxes) (n >= 1).

The last co-expression strategy tested was the single transcript strategy comprised of several shRNA domains (or technically just dsRNA domains, depending on design). There are two basic configurations, the 'cluster' (CL) configuration and the 'head-to-tail' (HT) configuration. Using pSilencer based vectors, we constructed two cluster arrays of three 29 bp hairpin domains (**R_V_T_1 _**and **R_V_T_8_**) with either 1 or 8 nt. spacers separating each domain (Table 2), and tested them with our fluorescent reporters (Figure 3a). Despite reported success from others [43,44], we only saw good activity in the first domain, with poor activity in the remaining two.

**Table 2:** Multiple domain, single transcript sequences

**Comb**.	Sequence ^a^	**Len**.
**R_V_T_1_**	GGAACUUAAGAGUGAAGCUGUUAGACAUU**ACUCGAGA**AAUGUCUAACAGCUUCACUCUUAAGUUCC**G**UAUAUUUCAAGGAAAGCUAAGGACUGGUU**ACUCGAGA**AACCAGUCCUUAGCUUUCCUUGAAAUAUA**G**AAACUGCUUGUACCAAUUGCUAUUGUAAA**ACUCGAGA**UUUACAAUAGCAAUUGGUACAAGCAGUUU**GUx**	204
**R_V_T_8_**	GGAACUUAAGAGUGAAGCUGUUAGACAUU**ACUCGAGA**AAUGUCUAACAGCUUCACUCUUAAGUUCC**GCUGCAGG**UAUAUUUCAAGGAAAGCUAAGGACUGGUU**ACUCGAGA**AACCAGUCCUUAGCUUUCCUUGAAAUAUA**GGACGUCG**AAACUGCUUGUACCAAUUGCUAUUGUAAA**ACUCGAGA**UUUACAAUAGCAAUUGGUACAAGCAGUUU**GUx**	218
**V-T**	**G**UAUAUUUCAAGGAAAGCUAAGGACUGGUU**GCUGCAGG**AAACUGCUUGUACCAAUUGCUAUUGUAAA**ACUCGAGA**UUUACAAUAGCAAUUGGUACAAGCAGUUU**GGACGUCG**AACCAGUCCUUAGCUUUCCUUGAAAUAUA**Ux**	144
**O'-O"**	AAGACAGUCCAACACACGCCACCUGUCUC**GCUGCAGG**AACAGUCUGUCAAA GGUGACCCCUGUCUC**ACUCGAGA**GAGACAGGGGUCACCUUUGACAGACUGUU**GGACGUCG**GAGACAGGUGGCGUGUGUUGGACUGUCUU**GUx**	144
**T-V**	**G**AAACUGCUUGUACCAAUUGCUAUUGUAAA**GCUGCAGG**UAUAUUUCAAGGAAAGCUAAGGACUGGUU**ACUCGAGA**AACCAGUCCUUAGCUUUCCUUGAAAUAUA**GGACGUCG**UUUACAAUAGCAAUUGGUACAAGCAGUUU**GUx**	145
**U-R**	**G**GCAAUGAGAGUGAAGGAGAAGUAUCAGCA**GCUGCAGG**GGAACUUAAGAGUGAAGCUGUUAGACAUU**ACUCGAGA**AAUGUCUAACAGCUUCACUCUUAAGUUCC**GGACGUCG**UGCUGAUACUUCUCCUUCACUCUCAUUGC**GUx**	145
**R-U**	**G**GGAACUUAAGAGUGAAGCUGUUAGACAUU**GCUGCAGG**GCAAUGAGAGUGAAGGAGAAGUAUCAGCA**ACUCGAGA**UGCUGAUACUUCUCCUUCACUCUCAUUGC**GGACGUCG**AAUGUCUAACAGCUUCACUCUUAAGUUCC**GUx**	145
**R-V-T**	GGAACUUAAGAGUGAAGCUGUUAGACAUU**GCUGCAGG**UAUAUUUCAAGGAAAGCUAAGGACUGGUU**GCUGCAGG**AAACUGCUUGUACCAAUUGCUAUUGUAAA**ACUCGAGA**UUUACAAUAGCAAUUGGUACAAGCAGUUU**GGACGUCG**AACCAGUCCUUAGCUUUCCUUGAAAUAUA**GGACGUCG**AAUGUCUAACAGCUUCACUCUUAAGUUCC**Ux**	217
**T-V-R**	AAACUGCUUGUACCAAUUGCUAUUGUAAA**GCUGCAGG**UAUAUUUCAAGGAAAGCUAAGGACUGGUU**GCUGCAGG**GGAACUUAAGAGUGAAGCUGUUAGACAUU**ACUCGAGA**AAUGUCUAACAGCUUCACUCUUAAGUUCC**GGACGUCG**AACCAGUCCUUAGCUUUCCUUGAAAUAUA**GGACGUCG**UUUACAAUAGCAAUUGGUACAAGCAGUUU**Ux**	217
**V-T_0_**	**G**UAUAUUUCAAGGAAAGCUAAGGACUGGUUAAACUGCUUGUACCAAUUGCUAUUGUAAA**ACUCGAGA**UUUACAAUAGCAAUUGGUACAAGCAGUUUAACCAGUCCUUAGCUUUCCUUGAAAUAUA**Ux**	128
**V^19^_-_T^19^**	**G**GGAAAGCUAAGGACUGGUU**GCUGCAGG**CUGCUUGUACCAAUUGCUAU**ACUCGAGA**AUAGCAAUUGGUACAAGCAG**GGACGUCG**AACCAGUCCUUAGCUUUCC**Ux**	106

**^a ^**: sequences external to the target-matched dsRNA domains are shown in bold (e.g. loops, spacers, U_x _termination remnants).

Following this we created a head-to-tail configuration comprised of two 29 bp hairpin domains (**V-T**) separated by an 8 nt. spacer. Suppressive activity was measured for both domains simultaneously by using dual reporters (GFPsTat and AsRed1sVif), which showed that each domain was simultaneously active (Figure 3b). We also show that this result was target-specific, since a second control molecule similarly constructed from two 29 bp off-target hairpin domains (**O'-O"**) showed no suppressive activity (Figure 3c). We further transferred the **V-T **head-to-tail configuration into a our pLenti6 based plasmid and assayed it using HIV-1 (i.e. a shared target) (Figure 3d). The net suppressive activity was comparable to the activities of the corresponding individual shRNAs and the combined activities of both individual shRNAs when delivered via the equivalent multiple cassette strategy. Encouraged by this finding, we assembled and tested several more head-to-tail configurations, comprised of the same domains but in different order (**T-V)**, different domains (**U-R **and **R-U**), more domains (**R-V-T **and **T-V-R**), no spacers (**V-T_o_**), and shorter 19 bp domains (**V^19^-T^19^**). However, in no case did we achieve a similarly successful outcome to our original **V-T **molecule (Figure 3e-h). While some domains were active, others were not, and no configuration retained comparable activity to the corresponding component shRNAs for all domains. We conclude that while two hairpins combined in a head-to-tail configuration can be successful, reliably obtaining an active molecule requires a more detailed design (than simply connecting pre-existing hairpins) that will come from a better understanding of how these configurations are processed.

## Discussion

In this study we tested 3 different strategies for the simultaneous expression of multiple hairpins and showed that all could be effective. But, the multiple vector strategy is likely to be of limited use in gene therapy since it requires a unique vector per shRNA, with potential issues in ensuring that each cell receives all vectors (without which may facilitate the emergence of resistant strains). The single transcript strategy was effective in one instance, but since similar success was not reproduced with different domains (or configurations), it is also of limited use in its present form. However, the multiple expression cassette strategy was used successfully with up to 4 shRNAs, and was easy to assemble and expand with pre-selected shRNAs.

When hairpins were co-expressed at levels that saturated the RNAi process we found that the *individual *suppressive activity of each hairpin was progressively reduced with increasing numbers of hairpins co-expressed. This was equally applicable for all three hairpin co-expression strategies; multiple vectors, multiple cassettes, and multiple domains. Hairpin competition was evident in all cases except one, where the activity of the Vpu 158-29 hairpin was little affected when transiently expressed with 3 other hairpins from a 4 cassette vector. Although the reasons for this are unknown to us, we note that this hairpin was very active and so it may be that even as it competed with 3 other shRNA its suppressive impact was unaffected. Overall, we surmise that hairpins interact competitively for access to the RNAi machinery. Whilst this conclusion is supported by some [45-47], it is worth noting that there are also conflicting conclusions, where others report no evidence of shRNA/siRNA competition [29,30,48,49]. The reason for this disparity may relate to differences in experimental design such as expression levels and observations under sub-saturating conditions. It should also be noted that issues of shRNA competition in mammalian cells encompass endogenous RNAi substrates as well, e.g. microRNA. Expression levels in a clinical setting may need to be finely tuned to attain sufficient activity, with minimal impact [2,50-53]. Another idea for removing competition may be to employ multiple agents of different modalities (e.g. RNAi, aptamers and ribozymes) so that no single pathway is overwhelmed [54,55].

When each hairpin of different sequence was directed to a common target (i.e. the complete HIV-1 sequence rather than individual gene-fusions), we saw that the *net *suppressive activity was approximately equivalent to the average activity of the component hairpins. This suggests that hairpin diversity may be increased whilst maintaining *overall *suppressive activity. This could potentially be exploited for countering the emergence of viral escape mutants in-line with other studies [27] though it requires further work for verification. Moreover, we did not test the effect on *net *activity of using one or more hairpins which was poor, or completely inactive (as all our hairpins here were classed as highly active). Such a situation could conceivably arise in a clinical setting due to a virus developing a mutation in one of the target sites. We speculate that the net suppressive activity would be reduced, though our mathematical modeling of various infection scenarios indicates that *some *loss of shRNA efficacy can be tolerated without impacting on treatment success [21,22].

Our data shows that up to 4 repeats of the same shRNA can increase the net suppressive activity when transiently expressed at levels below that which results in maximal suppressive activity. Interestingly, the same effect was not seen in the corresponding stably transduced cell lines. The reasons for this are unclear, but could result from promoter interference [41], or transcriptional silencing [56]. Other studies have shown that promoter interference is not necessarily a barrier to multiple shRNA cassette strategies [31,32,54]. One study has shown that up to 6 identical expression cassettes could increase total expression and suppressive activity during both transient and stable expression, though in this case more than six cassettes was deleterious in stable expression as it decreased the net suppressive activity [32]. Repeat-mediated cassette deletion is also a concern, as we and others have since shown that it commonly occurs during infection [21,51], possibly via reverse transcriptase slipping on its template [57], though again, our modeling suggests that the practical impact of this in a gene therapy setting may be low [21]. Finally, the reduction in titre that we observed, whilst inconsequential here, has also been noted by others [42,58-60] and is an issue that may have to be addressed prior to scaled-up manufacturing.

Our attempts to generate a cluster of 3 shRNA domains in a single transcript with activity retained equally in each domain were unsuccessful. However, due to the highly structured templates, we were unable to confirm the sequence of these templates with automated sequencing procedures. Thus we cannot rule out the possibility of single nucleotide errors in these configurations. This is another issue that needs to be overcome, though Liu *et. al*. have reported success by incorporating several G:U wobbles [61]. Head-to-tail configurations can be sequenced though (using a modified protocol [62]), and in this respect may be a more attractive choice. However, since these are akin to 'long' hairpins, they in turn may induce non-specific dsRNA-response pathways such as protein kinase R (PKR) or interferon (IFN) [63,64], though recent work suggests otherwise [61]. Even though our designs incorporated spacers (of 1 or 8 nt.) to keep all regions of paired double-stranded RNA less than 30 bp (the minimal length traditionally thought to activate non-specific pathways), there are reports that some structures outside of this traditional view (e.g. < 30 bp) may also stimulate these responses [65-68]. At this point though, the principle limitation of these configurations is in not knowing how they are processed, and consequently how they should be designed to reliably retain activity in all domains. Based on our understanding of single shRNA processing, where a single nt. shift in the start of the shRNA stem can significantly alter activity, we speculate that the processed products from our multiple domain constructs are simply different from the those liberated from the 'corresponding' single shRNAs. Liu *et. al*. and others are making inroads in this area [61,69], which will be well complemented by future deep-sequencing type studies. It is interesting to note that others who have also looked at similar structures have been similarly unable to produce effective silencing from more than 3 domains [61]. As a workaround one group has stacked several 2-domain structures, which could then be further used in combination with a multiple cassette type arrangement to increase targeting capacity [39].

In summary, we found that while all 3 co-expression strategies tested were effective, the multiple cassette strategy is a most useful method for immediate use in gene therapy. This is because it was easy to design, assemble, and is directly compatible with pre-existing shRNA already selected for high activity. It is worth noting that a similar study was published during the preparation of this manuscript, with similar conclusions, thus strengthening the validity of our findings [70]. Furthermore, we have since applied the multiple cassette strategy in several additional studies, including the development of a repeating modular cloning method (tested with up to 11 shRNAs), the assembly of combinations of up to 7 shRNAs to target entire subtypes of HIV-1, and a large-scale study around repeat-mediated deletion of 1 or more cassettes [21,71].

## Methods

### shRNA design and vector construction

Each shRNA was designed so that the sense or upper strand of the shRNA stem was homologous to the target (designed to give rise to the siRNA passenger strand) and the anti-sense or lower strand of the shRNA stem was complementary to the target (designed to give rise to the siRNA guide strand) (Table 1). Sense and anti-sense strands were connected by an 8 or 9 nt loop and all hairpins were expressed from a human H1 polymerase III (pol III) promoter with transcription presumably terminating at a run of 4 or more 'T' residues in the included termination signal (TTTTTTGGA). Each shRNA insert was constructed using either annealed complementary oligonucleotides (oligos) or primer extension [62] to create a synthetic DNA insert that was cloned into a pSilencer 3.0-H1 derived vector (Ambion). The pSilencer derivative was generated by replacing the bla gene (ampicillin^r^) for the neo gene (kanamycin^r ^/G418^r^). Single cassette pLenti6 based vectors were created by sub-cloning entire shRNA expression cassettes from the pSilencer based vectors into a derivative of pLenti6/V5-D-TOPO (Invitrogen). The pLenti6 derivative was generated by exchanging the CMV promoter, V5 epitope and SV40 terminator region for a multiple clone site (MCS) to facilitate the unique insertion of 1 to 4 self-contained (i.e. consisting of promoter, hairpin and terminator) shRNA expression cassettes. Multiple cassette pLenti6 based vectors were created by PCR amplification of the desired cassette(s) from the corresponding pSilencer based vector(s), which were then progressively inserted into the appropriate pLenti6 based vector downstream of any previous cassettes with a gap of ~ 130 bp separating each cassette. pSilencer based vectors were propagated in GT116 *E. coli *cells (a cell line specifically developed for the replication of hairpin containing vectors, Invivogen) and pLenti6 based vectors were propagated in Stbl3 *E. coli *cells (manufacturer recommended cell line, Invitrogen). DNA was extracted (Hi-speed Maxi-prep Kit, Qiagen), quantitated in triplicate and was sequence confirmed either by standard protocols or a modified protocol where required to enable automated sequencing of hairpin expression vectors possessing reaction-inhibiting secondary structure [62] (excluding the multiple hairpin 'cluster' configurations as indicated in the text).

### Reporter vector construction

The fluorescent protein-target fusion reporter vectors, were constructed using EGFP (from pd4-d4EGFP-N1, BD Biosciences), AsRed1 (from pAsRed1-C1, BD Biosciences) or AmCyan (from pAmCyan-C1, BD Biosciences) and HIV-1 sequences (variant NL4-3, accession #AF324493). Each vector was designed to produce a single mRNA transcript comprising the fluorescent protein fused to a downstream HIV-1 gene sequence but separated by multiple stop codons to ensure that only the first domain would be translated (the fluorescent protein). The sizes of reporter vectors deviated by no more than 10%. The reporters used here included: GFPs**T**at (T.r) and AsRed1s**V**if (V.r-red), GFPs**V**if (V.r), GFPsVp**r **(R.r) and GFPsVp**u **(U.r), and AsRed1s**N**ef (N.r-red).

### Fluorescence based shRNA activity assay

Human Embryonic Kidney cells (HEK293a, sourced from the American Type Culture Collection) were seeded at a density of 4 - 5 × 10^5 ^cells/well in 6 well plates in 2 ml of Dulbecco's modified eagle medium plus 10% fetal bovine serum (DMEM-10). Cells were transfected 1 day later using 1 μg of total DNA (comprised of different amounts of shRNA and/or 1 or more reporter vectors as indicated in each figure) with 4 μl of Lipofectamine 2000 (Invitrogen) in OptiMEM (Invitrogen) to a total volume of 100 μl/well. Cells were analyzed by flow cytometry 2 days later (using either a FACsort or FACsCalibur instrument, BD Bioscience). The suppressive activity of each shRNA was measured as a change in fluorescence of the reporter(s) (FL1 for 'green' proteins and FL2 for 'red' proteins). The Fluorescence Index (FI) of cells in each channel was calculated by multiplying the geo mean of fluorescence by the percentage of cells that were fluorescent (only those cells gated above background). The FI was expressed as a percentage of the FI of cells transfected only with the corresponding empty expression control vector that expresses no hairpin. Target-specific shRNA activities were normalized to account for non-specific effects measured using an additional 'green' or 'red' off-target reporter to which the shRNA bore no homology, except for cases where the simultaneous activities of 2 shRNAs where measured using a 'green' reporter for one shRNA, and a 'red' reporter for the other shRNA. Most assays included a 29 bp off-target control shRNA (**O'**), which displayed no meaningful suppressive activity against any reporter, and thus was omitted from the graphs for clarity.

### Lentivirus production and infection

293FT cells (Invitrogen) were seeded at a density of 5 × 10^6 ^cells/plate (100 mm plates; 10 ml DMEM-10) and were transfected 1 day later using 12 μg of total DNA (comprised of 3 μg pLenti6 based hairpin vector and 9 μg packaging vectors, Invitrogen) with 36 μl of Lipofectamine 2000 in OptiMEM to a total volume of 8 ml/plate. Virus-containing medium (VCM) was harvested at 2 - 3 days post-transfection, cold spun at 3000 rpm for 15 min. and stored at -80°C. Viral titres were calculated using HEK293a cells seeded at 1 × 10^5 ^cells/well (6 well plates; 2 ml DMEM-10) which were infected with serial dilutions of VCMs ranging from 10^-1 ^to 10^-6 ^supplemented with 6 μg/ml of polybrene (hexadimethrine bromide, Sigma). Selective medium (DMEM-10 plus 10 μg/ml Blasticidin, Invitrogen) was applied to infected cells 2 days later and maintained for 10 - 14 days prior to Giemsa staining (Merck) and quantification of colony numbers, with titres calculated as infectious viral particles (IVF)/ml of VCM. Stably transduced cell lines were generated using HEK293a cells seeded at a density of 4 × 10^5 ^cells/plate (6 well plates; 2 ml DMEM-10) which were infected 1 day later with 2 ml of VCM with an average MOI of ~ 0.4. Selective medium (DMEM-10 plus 10 μg/ml Blasticidin) was applied to infected cells 4 days later and maintained for at least 14 days.

### HIV-1 production assay

HEK293a cells were seeded at a density of 2 × 10^5 ^cells/well (12 well plates; 1 ml of DMEM-10). Cells were transfected 1 day later using 110 - 130 ng of hairpin expression vector (at equimolar amounts across transfections) and 800 ng (3× molar amount of expression vector) of pNL4-3 reporter vector (expressing the 4-3 strain of HIV-1) with Lipofectamine 2000 at a ratio of 1 : 4 (μg DNA: μl Lipofectamine) in OptiMEM to a total volume of 200 μl/well. Medium was replaced with an equal volume 1 day post-transfection and the cells were harvested a further 1 day later by centrifugation at 400 g for 10 min. at room temperature. Samples were stored at -20°C until assayed for p24 levels (a capsid protein required for HIV-1 virion production) via Enzyme-Linked Immunosorbent Assay (ELISA) using the INNOTEST HIV antigen mAb kit (Innogenetics). The suppressive activity of each shRNA was measured as a reduction in, and expressed as percentage of, p24 production (measured as pg/ml) relative to p24 production from cells transfected with the corresponding empty expression control vector.

### Statistical analysis

Each sample was analyzed in triplicate with 95% confidence intervals (CI) calculated using Microsoft Excel X. P values were determined by analysis of variance (ANOVA, with a Bonferroni's multiple test comparison) using Prism 4.0a.

## Competing interests

This work was done jointly by GJM, at the time a student enrolled in The school of Biotechnology and Biomedical Science at the University of New South Wales, Sydney, Australia, and employees of Johnson and Johnson Research (JJR), for Johnson and Johnson Research.

## Authors' contributions

GJM and GCF conceived the experiments. GJM designed the constructs, made the plasmids and performed the fluorescence experiments. AJA, KMG, and WMM performed the Lentiviral production and HIV expression experiments. GJM wrote the manuscript. All authors have read and approved the final manuscript.
